# Research on the Mechanism and Processability of Roll Forming

**DOI:** 10.3390/ma17133126

**Published:** 2024-06-26

**Authors:** Cunfeng Kang, Baoxu Sun, Xinshang Zhang, Chengxi Yao

**Affiliations:** College of Mechanical and Energy Engineering, Beijing University of Technology, Beijing 100124, China; sunbaoxu@emails.bjut.edu.cn (B.S.); zhangxxinshang@emails.bjut.edu.cn (X.Z.); yaochengxi@emails.bjut.edu.cn (C.Y.)

**Keywords:** high-strength steel, thin-walled circular tube, cold roll forming, edge extension, equivalent plastic strain

## Abstract

Cold bending forming is a complex forming process, and its product quality is closely related to the forming process parameters. To mitigate issues such as bulging and waviness arising from the extension of the material at the edges during the forming process of thin-walled circular tubes, a comprehensive comparative analysis was conducted on four forming methods. This analysis determined that the combined bending method is the optimal forming technique for the equipment. For the impact of different parameters on the equivalent plastic strain distribution of the product and the force on the rollers, numerical simulations were carried out using the software COPRA (COPRA FEA RF 2023.1) after designing the pattern diagram based on the integrated bending method. The results showed that different processing speeds on the equivalent plastic strain distribution and work hardening of the plate have little effect. As the spacing between the upper and lower rollers increases, the equivalent plastic strain of the plate to a certain extent and the value of the moment of the rollers is significantly reduced. Analyzing the performance characteristics of high-strength steel materials from the aspects of the thickness strain and cross-sectional forming of the plate, this verifies the advantages of forming high-strength steel plates. The numerical simulation results of this study are in good agreement with actual production experimental results.

## 1. Introduction

With the advancement of modern industry, the importance of thin-walled circular tubes in various applications is increasingly being recognized. Currently, thin-walled circular tubes are essential raw materials for Internal High-Pressure Forming (IHPF). Thin-walled tubes have found wide application in industries such as automotive manufacturing and aerospace [1,2]. The study of welded pipe forming technology is of significant importance for energy conservation, environmental protection, and the development of lightweight vehicles [3]. 

Cold roll forming (CRF) is a process using a series of rolls to bend a sheet blank into the desired cross-sectional profile continuously [4]. The layout and shape of the adjacent rolls is called a roll flower. Forming defects, such as bowing, sweeping, twisting, flaring, and cross bowing, may occur if the roll flower is not designed suitably. Due to the inherent characteristics of the continuous roll forming process, the edges of the material extend during bending. If this extension rate does not exceed the material’s elastic stretching limit, it will elastically recover in the later stages of forming, not affecting the quality of forming and welding [5,6]. However, irreversible plastic extension can lead to bulging and waviness at the edges of the material, decreasing the quality of forming and welding and, in some cases, making welding impossible [7,8].The equivalent plastic strain produced during welded pipe forming is closely related to the number of roll passes. If the distribution of equivalent plastic strain in the material is uneven during roll forming, it can severely affect the subsequent high-pressure forming process and may even lead to bursting of the tube. Therefore, in the design of the thin-walled circular tube forming process, controlling the edge elongation of the strip within the range of elastic deformation is crucial to ensure forming quality [9,10]. Additionally, improving the uniformity of work-hardening distribution to enhance the processability of high-pressure forming is the main issue we aim to address.

The optimal configuration of roll forming process parameters, such as the operational line speed, the inter-distance between roll stations, the roll gap, and the diameter of the rolls, can influence the tooling and product designs as well as the product quality. Li et al. [11] conducted a finite element simulation study on the chain die forming of U profiles with variable cross-sections, including variable width and height. The successful simulation results verified the feasibility of using chain die forming to produce products with variable cross-sections. Kasaei et al. [12] studied the flange wrinkling at the transition zone using finite element analysis and developed a flexible roll forming set up. Rossi et al. [13] used the finite element analysis software METAFOR to compare the forming process from high-strength and stainless steels, where the effect of different bending angle to thickness ratios on the longitudinal strain of the plate was taken into account in the simulation. Lindgren et al. [14] analyzed the roll forming process of U-grooves made of high-strength steel and found that as the yield limit of high-strength steel increases, the peak longitudinal strain decreases, and the deformation length increases. Paralikas et al. [15,16] determined the optimal parameter configuration of the roll forming line through experimental simulation and found that the equivalent plastic strain distribution of the formed profile and the roll spacing are the main factors affecting the forming effect. Murugesan et al. [17] investigated the presence of longitudinal bow, the reason behind the flange height deviation, spring-back, and identification of the thinning location in the cold roll forming of symmetrical short U-profile sheets. Wang et al. [18] investigated the effects of roll spacing, friction coefficient, diameter increment, and linear velocity on the maximum longitudinal strain at the edges by using the orthogonal experimental method and proposed a floating roll device suitable for asymmetric large-depth complex sections. 

Based on the optimization of the aforementioned process parameters, most studies overlook the impact of the material properties of the steel itself. High-strength steel, due to its superior hardenability and improved Charpy V-Notch (CVN) impact toughness, is widely used in the manufacture of straight seam welded pipes [19]. The roll bending forming technology for high-strength steel straight seam welded pipes represents a modern manufacturing technique, primarily applied in producing high-strength, high-precision steel tubes and components [20]. Although this technology has been widely adopted domestically, there remain certain challenges. In the aspect of roll bending forming process, the forming difficulty of high-strength steel materials is relatively high, necessitating the use of higher roll bending pressures and smaller bending radii to determine the optimal forming method. Moreover, during the roll bending forming process of high-strength steel materials, issues such as excessive residual stress and uneven deformation often arise [21]. Accurately studying the stress distribution and deformation patterns is of significant importance for improving the quality and efficiency of roll bending forming [22]. Therefore, research into the roll bending forming technology for high-strength steel straight seam welded pipes needs to explore and solve technical problems from multiple aspects to further enhance the quality and efficiency of roll bending forming.

This paper calculates and compares the length and height of the edge movement of the sheet in the process of four kinds of tube curling forming methods: circumferential bending method, edge bending method, center bending method, and combined bending method. In consideration of actual production requirements, the advantages and disadvantages of four forming methods and their effects on the extension of plate edges were analyzed to determine the optimal forming method. Based on the combined bending method, the bending radius and angle of each pass were calculated, and then, the roll flower diagram was drawn. A finite element numerical simulation of the whole process of roll forming of thin-walled round tubes of high-strength steel was conducted based on the pattern diagram. Addressing the challenges of actual production speed requirements and work hardening, the equivalent plastic strain distribution of thin-walled round tubes of high-strength steels during the forming process was further investigated. Utilizing the single-variable method, the impact of two critical parameters, production speed and the gap between the upper and lower rolls per pass, on forming quality and roll force was determined, with the research findings validated through practical adjustments and testing. According to the numerical simulation rules, the roll forming production line can be optimized, the service life of the equipment can be prolonged, and guidance suggestions for roll forming of high-strength steel sheets can be provided. 

The optimization of the molding process can enhance the strength and stiffness of thin-walled round tubes, enabling them to withstand the same load with a thinner wall thickness. The resulting improvement can be utilized in the automotive manufacturing and aerospace lightweight development industries.

## 2. Materials and Methods

### 2.1. Materials

CR700/980DP steel is a material of choice for the manufacture of automobile parts due to its high strength and good cold deformation properties. CR700/980DP metal material are selected as the material model data used in the plate as shown in Table 1.

### 2.2. Choice of Forming Strategy

#### 2.2.1. Analysis of Material Edge Extension

The ratio of wall thickness to outer diameter in thin-walled tubes is relatively small, leading to poor stability at the edges. Under the same working conditions, wrinkling becomes more pronounced when the wall thickness is less than or equal to 5% of the outer diameter. Therefore, for high-strength steel thin-walled tubes, specifically those with a wall thickness to outer diameter ratio of 5% or less, the main factor influencing forming quality is edge elongation.

In the forming of thin-walled tubes of high-strength steel, the edge elongation of the sheet metal is influenced by factors such as forming method, deformation zone length, amount of downward movement during forming, and subsequent corrective modules. In this study, an appropriate forming strategy was selected based on theoretical calculations of cross-sectional geometric changes during the sheet metal forming process and a comprehensive analysis of empirical experiences in actual forming processes.

The rolling process was complex because in roll forming methods, the rolls were not integral, and there were gaps between each set of rolls. Therefore, in practical forming processes, the stress on the sheet metal occurred in segments. During this segmented forming process, significant mechanical changes occurred at the edge of the sheet metal. Additionally, the different roll profiles resulted in different amounts of edge elongation. Due to the complexity of production conditions involved, it is challenging to quantitatively calculate edge elongation.

However, under ideal conditions without considering spring-back effects, for different forming strategies when determining deformation zone length, theoretically comparing trajectory equations for points along the edge can provide an estimate for edge elongation. The longer trajectory length indicates a greater stretch during the formation process and, hence, a larger theoretical value for edge elongation [23].

In the forming zone, the edge elongation is directly proportional to the square of the height of the deformation zone (i.e., the sheet thickness) and inversely proportional to its length (i.e., the sheet width). Since the edge elongation of a steel tube is related to both its height and width during the forming process, we can control the rise height and trajectory length of the sheet metal at the edge to reduce edge elongation.

#### 2.2.2. Comparison of the Geometric Deformation of Different Forming Strategies

**Figure 1:**
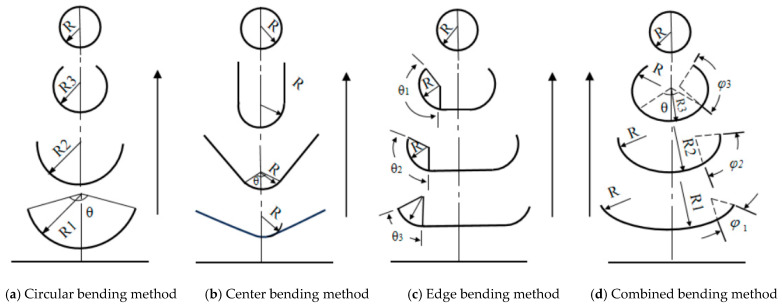
Schematic of four forming methods.

The curling process can vary significantly, and selecting the appropriate geometric forming method for high-strength steel sheet is crucial in reducing edge elongation during the forming process. For high-strength steel tubes, there are four commonly used forming methods: circumferential bending, center bending, edge bending, and comprehensive bending, as shown in Figure 1.

By using mathematical calculations, we can analyze and compare these four forming methods in terms of rise height, lateral distance traveled by the sheet metal edge during forming, and trajectory [24].

The circular bending method is shown in Figure 1a. The trajectory equations are given in the following at the edge of the forming plate:

(1) $$Y=\frac{\pi R}{\theta }*(1-\cos\theta ),$$

(2) $$X=\frac{\pi R}{\theta }*\sin\theta ,$$

(3) $$Y_{\max}=2.28R,$$

(4) $$L=\int_{0}^{\pi }\sqrt{dx^{2}+dy^{2}}dxdy=\frac{\pi D}{2}\int_{0}^{\pi }\left[\sqrt{1+\frac{2}{\theta^{2}}-\frac{2\sin\theta }{\theta }\frac{2\cos\theta }{\theta^{2}}}\right]d\theta =4.44R.$$

where Y is the plate material edge rising height, X is the horizontal displacement of the plate edge, D is the diameter of forming tube, θ is the formed arc corresponding to the center angle, R is the forming radius of the arc, φ is the circular corner of the welded pipe radius in the integrated forming method, $Y_{\max}$ is the plate edge rise height maximum, and L is the projection of the trajectory of the plate edge.

The central bending method is shown in Figure 1b. The trajectory equations are given in the following at the edge of plate shaped material:

(5) Y=R[1−cosθ+(π−θ)sinθ],

(6) X=R[sinθ+(π−θ)cosθ],

(7) $$Y_{\max}=(1+\frac{\pi }{2})R=2.57R,$$

(8) $$L=\int_{0}^{\pi }\sqrt{dx^{2}+dy^{2}}dxdy=\frac{D}{2}\int_{0}^{\pi }\sqrt{\left(\pi -\theta \right)}d\theta =4.49R.$$

Edge curve path equations for edge running plate are shown here:

(9) Y=R(1−cosθ),

(10) X=R[(π−θ)+sinθ],

(11) $$Y_{\max}=2R,$$

(12) $$L=\int_{0}^{\pi }\sqrt{dx^{2}+dy^{2}}dxdy=\frac{D}{2}\int_{0}^{\pi }\sqrt{2(1-\cos\theta )}d\theta =4.0R.$$

For the combined bending method, the trajectory equation is determined by φ and two variables, so it is impossible to obtain an accurate edge trajectory equation. But the changes in edge direction can be listed, respectively:

(13) $$Y=R(\frac{\pi -\varphi }{\theta }(1-\cos\theta )+\cos\theta -\cos(\theta +\varphi )],$$

(14) $$X=R[(\frac{\pi -\varphi }{\theta }-1)\sin\theta +\sin(\theta +\varphi )],$$

From (13) and (14), the change in Y and X direction is unrelated to φ and θ. Without determining the specific relationship between them, it is not possible to calculate precise values. However, since the combined bending method is a combination of circumferential bending and edge bending methods, we can infer the relationship between the rise height (Y) and lateral distance traveled (L) at the edge. For roll forming of high-strength steel, the $Y_{\max}$ of the combined bending method is 2.0R~2.57R, and the maximum running locus value is 4.0R~4.44R.

The numerical calculation of the sheet metal trajectory equation shows that the edge bending method has the smallest length (L) of sheet metal edge trajectory and the smallest lift height (Y), followed by the combined bending method.

From the analysis of the forming process, it can be concluded that the circular bending method is a relatively simple process that results in uniform deformation. However, due to the shape of this forming roller, it is prone to misalignment in sheet metal motion, causing twisting at weld seam positions. Therefore, auxiliary devices are used to prevent twisting during use. Both center bending and edge bending methods bend depend on the radius of curvature for forming steel tubes, wherein the roller designs are simple and provide stable forming processes. However, when precision shaping is required, there may be a lack of bending guidance for unbent sections that require multiple bends to ensure consistent quality. The combined bending method combines advantages from both circumferential bending and edge bending methods by providing stability during formation with good results. Simultaneously folding both edges and middle sections reduces overall bend count, as summarized in Table 2.

For the roll forming characteristics of high-strength steel, we consider the limit of edge elongation, forming stability, and bending times comprehensively and also analyze the forming process and edge movement track length of sheet bending comprehensively. It is best to choose the combined bending method to achieve a stable forming process with minimal edge elongation.

### 2.3. Forming Analysis of Thin-Walled Circular Tube

#### 2.3.1. Sheet Metal Roll Pattern and Forming Unit Design 

Based on the above analysis, the composite bending forming method combines the advantages of edge bending and circumferential bending methods, resulting in a stable forming process where the material edges are sufficiently deformed, leading to high-quality steel tube formation. Hence, the design of the forming mill in this paper is based on an analysis using the combined bending method. 

Assuming that using a cubic curve to represent the projection trajectory of the pipe edge onto the horizontal plane is the most appropriate allocation of bending angles for the sheet material, the bending radius $\rho_{ci}$ and angle $\theta_{ci}$ values for any pass are to be determined. Let the total number of forming passes be denoted by N and the width of the raw material sheet be denoted by L. The expression for the cubic curve and the boundary conditions are given by Equations (15)–(17), respectively, with the assumption that Equation (18) holds.

(15) $$y=Ax^{3}+Bx^{2}+Cx+D$$

When x=0 and x=N,

(16) $$\frac{dy}{dx}=0$$

When x=0,

(17) $$y=\frac{L}{2}$$

When x=N,

(18) y=0

The projection length y from the edge of the pipe to the center horizontal plane of the pipe for the i th pass can be determined by the following equation:

(19) $$Y_{i}=\frac{L}{2}\left[2{\left(\frac{i}{N}\right)}^{3}-3{\left(\frac{i}{N}\right)}^{2}+1\right]$$

For the roller design of the combined bending method, we need to establish two boundary conditions:

(1) Bending is performed by the edge forming method from the edge of the sheet to $\frac{\pi D}{4}$ (D is the diameter of the pipe), and by the circular forming method from $\frac{\pi D}{4}∼\frac{\pi D}{2}$.

(2) The bending amount of each pass in the above edge forming method is evenly distributed.

**Figure 2:**
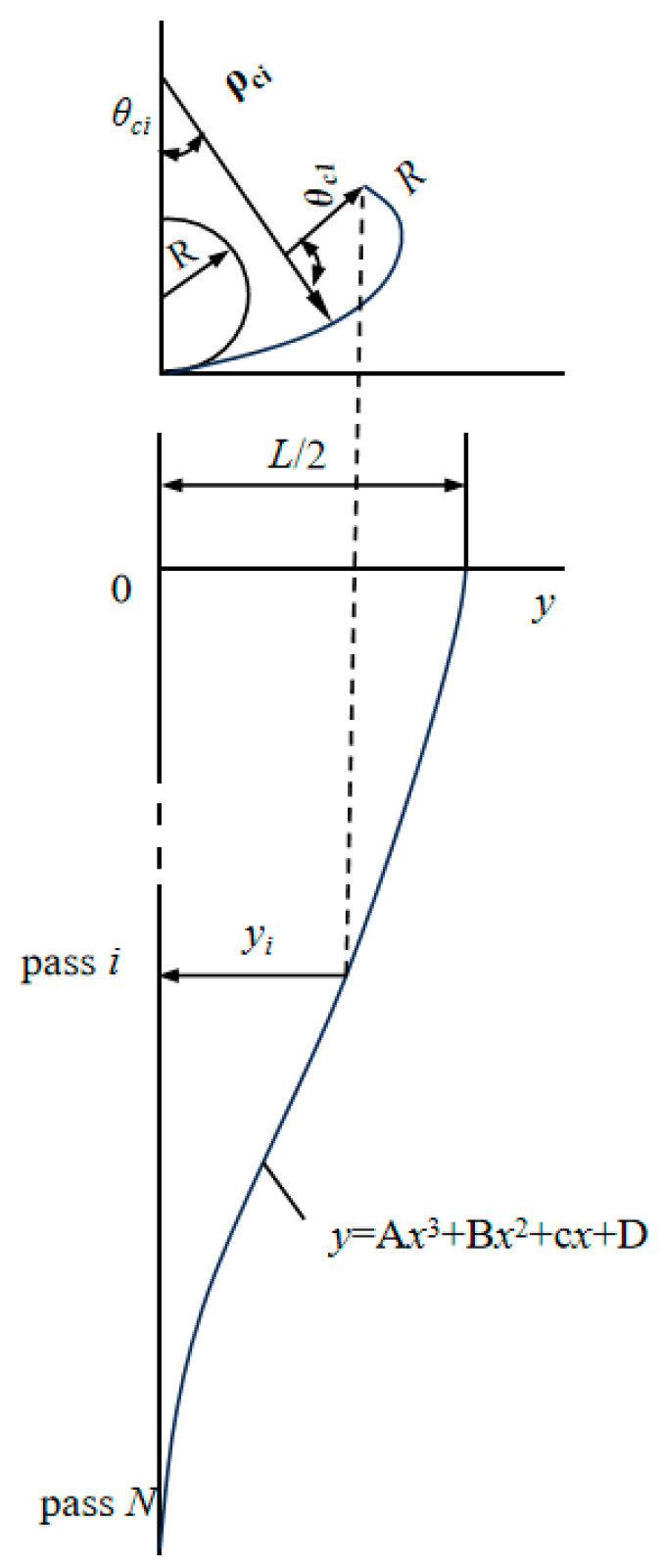
Schematic diagram of the molding principle of the combined bending method.

As shown in Figure 2, the bending radius $\rho_{ci}$ and angle $\theta_{ci}$ for the i pass using the circular forming method, the bending radius R and angle $\theta_{ei}$ for the edge forming method, and the bending radius R and angle $\theta_{e1}$ for the edge forming method in the 1st pass are specified.

Based on $y_{i}=(\rho_{ci}-R)\sin\theta_{ci}+R\cos\left(\theta_{ci}+\theta_{ei}-\frac{1}{2}\pi \right)$, the following equation can be obtained:

(20) $$\rho_{ci}\theta_{ci}=\frac{L}{2}-R\theta_{ei}$$

(21) $$\theta_{ei}=\theta_{e1}+\left(\frac{i-1}{N-1}\right)\left(\frac{\pi }{2}-\theta_{e1}\right)$$

The horizontal projection length $y_{i}$ from the edge of the pipe to the center of the pipe for the i th pass is determined from the geometric relationship in the figure, as shown in Equation (17).

(22) $$y_{i}=(\rho_{ci}-R)\sin\theta_{ci}+R\cos\left(\theta_{ci}+\theta_{ei}-\frac{1}{2}\pi \right)$$

Substituting the $\rho_{ci}$ in Equation (20) into $\rho_{ci}$ in Equation (22) yields Equation (23).

(23) $$y_{i}=\left(\frac{\frac{L}{2}-R\theta_{ei}}{\theta_{ci}-R}\right)\sin\theta_{ci}+R\cos\left(\theta_{ci}+\theta_{ei}-\frac{\pi }{2}\right)$$

When given an initial value for $\theta_{ei}$, fitting $Y_{i}$ and $y_{i}$ using the bisection method, with an error requirement smaller than $10^{-5}$, we can obtain the roller parameters. Furthermore, introducing a variation coefficient m, we consider the equation of the projection curve as follows:

(24) $$Y_{i}=\frac{L}{2}\left[2{\left(\frac{i}{N}\right)}^{3+m}-3{\left(\frac{i}{N}\right)}^{2+m}+1\right]$$

**Figure 3:**
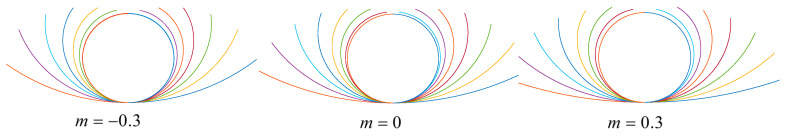
The roll flower pattern diagram for circular pipes obtained with different variation coefficients m.

We observe that, as shown in Figure 3, when the variation index is set to −0.3, the latter part of the forming process is relatively refined. Conversely, when the variation index is set to +0.3, the former part of the forming process is relatively refined.

**Figure 4:**
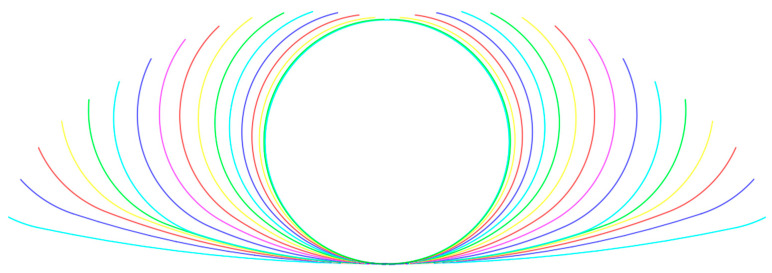
The flower pattern of tube roll forming diagram.

According to the above formulae, to ensure uniform deformation and considering the characteristics of the high-strength steel material selected for the sheet, the parameter m is determined. A forming flower pattern diagram is plotted using MATLAB (2021), with specific parameters as shown in Table 3. The roll pattern of the material refers to the state of the material’s unfolding at different forming passes, representing the transition from a flat plate to the required section profile, indicative of the technological level of roll bending forming, as shown in Figure 4. During the roll bending forming process, the material undergoes plastic deformation under the action of the rolls. The pattern provides a visual representation of the material’s state during bending, identifying potential flaws or deformations that may occur. Prompt measures are taken for adjustment and correction to ensure the quality of the finished product.

**Figure 5:**
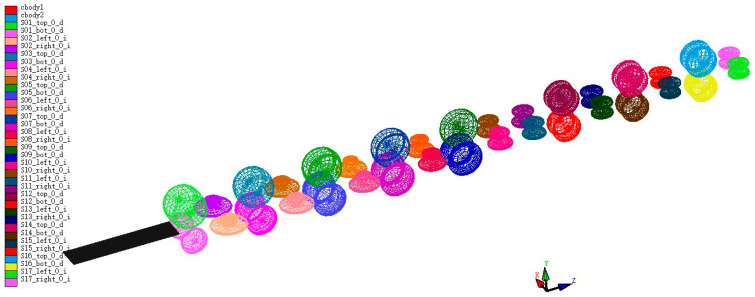
Model of the forming roll mill unit.

**Figure 6:**
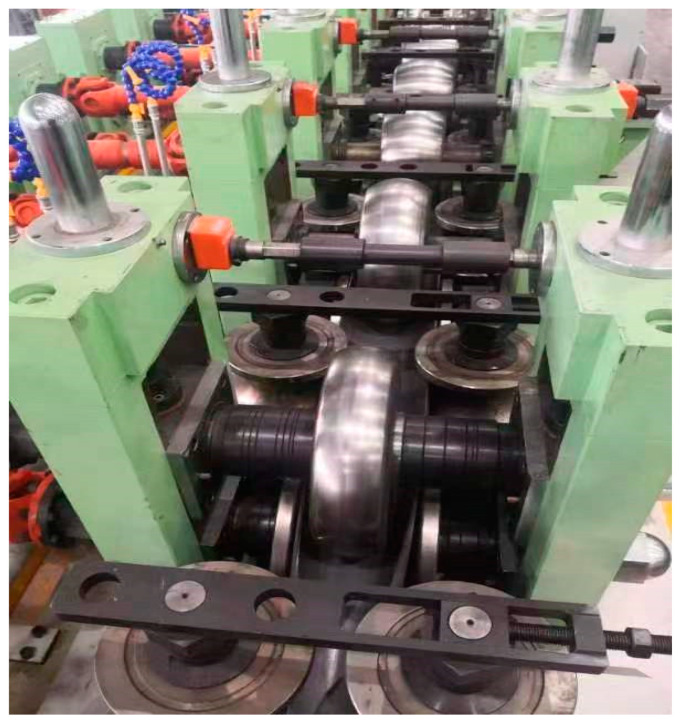
Forming roll mill unit.

A circular tube roll forming mill generally includes pre-forming, line-forming, and precision-forming sections, as shown in Figure 5, allowing the material to ultimately achieve a circular cross-section. During the initial loading process, a storage loop provides power, pushing the material into the pre-forming stage, where the main power is supplied by the forming mill, with the first set of rolls serving as guide rolls. The forming mill adopts a semi-continuous roll forming method, which is more suitable for the manufacturing of thin-walled circular tubes compared to the continuous roll forming. For manufacturers producing large volumes of the same specification, this forming method is simple, energy-efficient, and low in equipment costs. The forming process follows a 5 + 2 + 3 formation, namely, 5 sets of pre-forming rolls (UO flat rolls), 2 sets of line-forming rolls (vertical rolls), and 3 sets of precision-forming rolls (UU flat rolls), with vertical rolls acting as auxiliary wheels between the flat rolls in both the pre-forming and precision-forming stages to assist the line-forming part, as shown in Figure 6.

#### 2.3.2. Model Analysis of the Material Forming Stage

Based on the dimensions and arrangement of rolls in the composite bending forming process, the forming stage of ϕ86 straight beam welded steel pipes are modeled using COPRA, with each pass’s roll forming radius consistent with the roll pattern. The material dimensions used are 1147 × 280 × 5 mm. 

**Figure 7:**
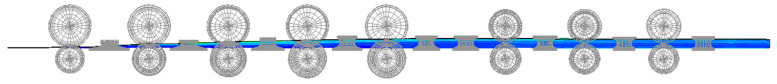
The schematic of the finite element model for the forming section.

The boundary condition is set as follows: In the simulation, the forming unit can simplify the rolls to only rotate around the axis center, as illustrated in Figure 7, not moving in the x-y-z plane. The friction coefficient between the plate and the roller wheel is set to 0.1, and the plate is displaced along the z direction by the friction force of the roller wheel to simulate the actual working condition. 

**Figure 8:**
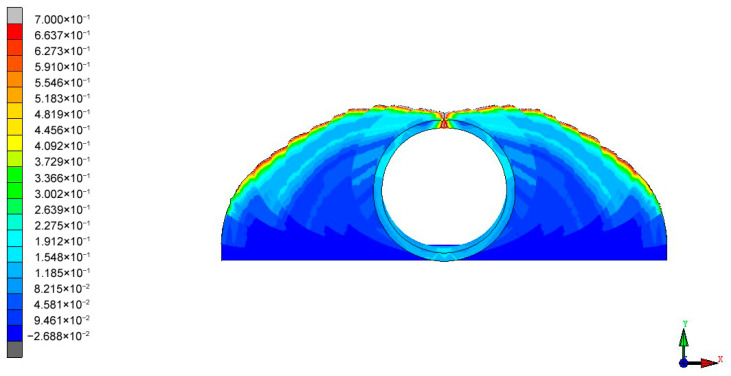
Cross-sectional equivalent plastic strain map of the material.

Based on the selected metal material, a simulation is performed using the forming roll group designed according to the aforementioned roll pattern, resulting in an equivalent plastic strain diagram of the sheet metal cross-section as shown in Figure 8.

### 2.4. Distribution of Equivalent Plastic Strain and Work Hardening Analysis in the Material

**Figure 9:**
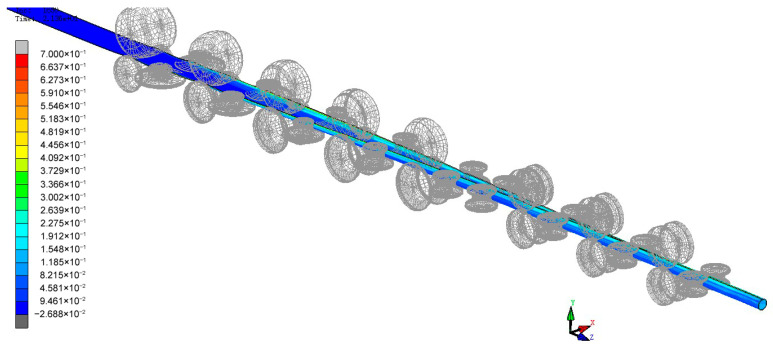
Overall equivalent plastic strain cloud diagram of the material.

**Figure 10:**
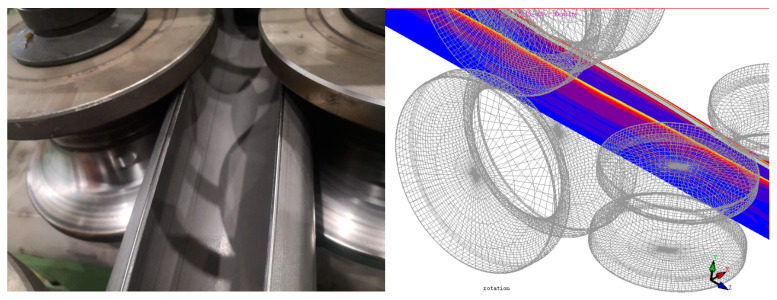
Comparison between physical and simulation of sheet metal forming and processing hardening.

The forming speed of the material and the gap between the rolls are important parameters during the rolling process, both significantly influencing the final forming quality of the steel tube. This paper first conducts a simulation analysis of the material curling at a real production tuning speed of 10 m/min and a gap between the upper and lower rolls of 5 mm. Figure 9 shows the equivalent stress cloud diagram of the material in the forming stages. From the equivalent plastic strain cloud diagram of the forming, it can be seen that the equivalent plastic strain of the material during the forming stage is mainly located at the edges and the contact parts between the rolls and the material. Thus, the main distribution of work hardening also occurs at the edges of the material and the contact parts, as shown in Figure 10. The work-hardening condition at the edges of the material is alleviated due to heating in the subsequent welding process. In actual situations, the failure of the weld seam of the circular tube mainly stems from welding quality issues. Therefore, the equivalent plastic strain and work hardening at the edge of sheet metal are not studied in this paper, and our greater focus is on the distribution of work hardening in other parts.

**Figure 11:**
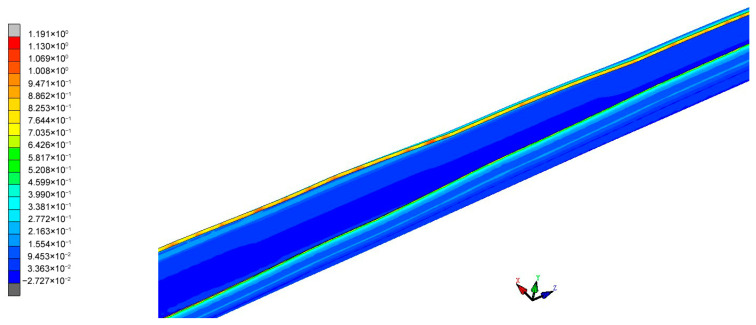
Cloud diagram of equivalent plastic strain detail in pre-forming stage.

Focusing on the distribution of plastic strain in the material, as shown in Figure 11, it is evident that the plastic strain of the material in the circumferential direction is zoned. And there is an obvious protruding region of equivalent plastic strain, and the plastic strain in the protruding region reaches 28%, indicating that the greater the value of equivalent plastic strain, the more severe the work hardening situation. Therefore, it is necessary to reduce the work hardening at this location to make it consistent with the surrounding areas; otherwise, burst failures might occur during high-pressure forming. Due to the different applications of the finished pipes or the varying stress conditions on different parts during use, understanding the work-hardening distribution of the formed pipes can better guide the production of these pipes and avoid unnecessary losses. 

To comprehensively analyze the strain distribution of the material during the forming process, we attempt to adjust the rolling speed and the gap between the upper and lower rolling rolls without changing the mold, for comparative simulation analysis. This aims to address the issue of pronounced work hardening in the welded tubes and to improve the quality of thin-walled circular tubes, extending the service life of the equipment.

## 3. Discussion

### 3.1. The Impact of Rolling Speed on Equivalent Plastic Strain during the Forming Stage

**Figure 12:**
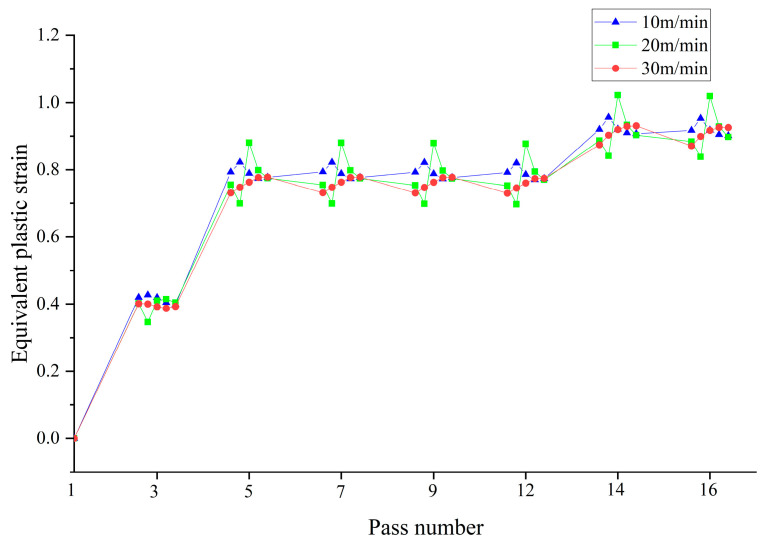
Equivalent plastic strain of material at different rolling speeds.

In modern factories, production efficiency is emphasized, and understanding the impact of rolling speed on equivalent plastic strain can effectively guide processing and production. As the production speed of circular tubes increases, the area that needs to be welded per unit time increases, requiring greater production power from the welding machine. Considering the rated power of the welding machine used in this project and the maximum speed of the flying saw, the maximum safe speed for the circular tube production line is set at 30 m/min. Therefore, this paper simulates the process at feeding speeds of 10 m/min, 20 m/min, and 30 m/min, as shown in Figure 12. Although the rolling speeds vary, the strain change pattern at the same node remains the same. When the rolling speed is 30 m/min, the maximum equivalent plastic strain of the material is 0.272; when the rolling speed is reduced to 20 m/min, the maximum equivalent strain increases slightly to 0.274, a mere 0.74% increase; and when the rolling speed is further reduced to 10 m/min, the maximum equivalent plastic strain increases to 0.276, a marginal increase of 0.73%. This indicates that keeping other parameters constant, when forming tubes with a diameter of 86 mm at production speeds of up to 30 m/min, reducing the rolling speed of the material has a minimal impact on its equivalent plastic strain. For processing safety considerations, this paper selects a rolling speed of 10 m/min to analyze the stress–strain distribution of the formed sheet metal.

### 3.2. The Impact of the Gap between Upper and Lower Rolls on Equivalent Plastic Strain

During the rolling process, the gap between the upper and lower rolls has a significant impact on the quality of material forming. In previous welded tube production processes, the gap between the rolls was equal to the wall thickness of the tubes. During rolling, changes in tube wall thickness can occur, with an increase in wall thickness potentially causing stress concentration or excessively high stress peaks, leading to more severe work hardening effects and affecting the quality of material forming. Therefore, the impact of the roll gap size on the forming outcome should be considered. 

**Figure 13:**
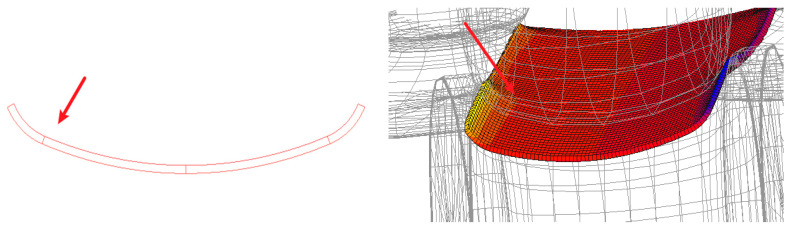
Schematic of selected node locations.

In order to make the distribution of work hardening more uniform, the position of the lower roller is fixed, and the position of the upper roller is changed to simulate without changing the die. Its effect on the equivalent plastic strain of the plate is studied. Because the plate passes through the intersection of two arcs with different centers in the combined forming method, the rolling deformation is the largest as indicated in Figure 13. The boundary is defined as the demarcation point, and the demarcation point of 8 passes is selected to discuss the relationship between the equivalent plastic strain and the gap between the upper and lower rollers. Under the condition of feed speed of 10 m/min, the working conditions of 5 mm, 5.3 mm, and 5.6 mm between upper and lower rollers are simulated, respectively. 

**Figure 14:**
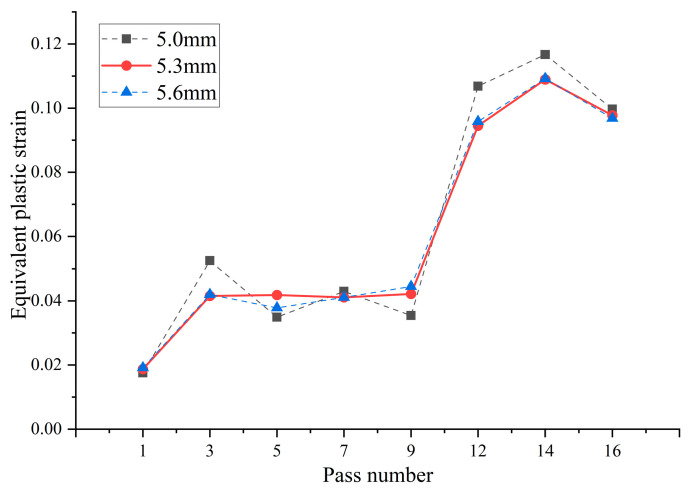
Equivalent plastic strain of material under different gaps between upper and lower rolls.

With varying gaps between the upper and lower rolls, the trend of equivalent plastic strain during the material forming stage changes with each pass, as shown in Figure 14. The overall equivalent plastic strain of the material shows an increasing trend, with the precision-forming stage having an average equivalent plastic strain 2.8 times that of the pre-forming stage. Despite the differences in the gaps between the upper and lower rolls in the three simulations, the maximum equivalent plastic strain during the pre-forming stage, specifically within the UO roll set, occurs in the third pass. The average equivalent plastic strain of the pronounced parts is 0.0524, 0.0415, and 0.0420, respectively, with the strain value for a 5.6 mm gap between the rolls decreasing by 19.8% compared to a 5 mm gap. In the precision-forming stage, specifically within the UU roll set, the fourteenth pass generates the maximum equivalent plastic strain. The average equivalent plastic strain of the pronounced parts is 0.1167, 0.1089, and 0.1092, respectively, with the strain value for a 5.6 mm gap between the rolls decreasing by 6.4% compared to a 5 mm gap. Therefore, it is evident that the equivalent plastic strain significantly decreases when the gap between the upper and lower rolls is set to 5.6 mm. This means that increasing the gap between the upper and lower rolls can, to some extent, reduce work hardening during the material forming process, making the plastic strain of the material more uniform and beneficial to improving the quality of the formed tubes.

### 3.3. The Impact of the Gap between Upper and Lower Rolls on the Force Experienced by the Rolls

In production, each set of rolls is driven by a separate servo motor. Changing the gap between the upper and lower rolls results in a noticeable change in motor torque values. Therefore, to balance the lifespan of the motors, the gap between the upper and lower rolls should not be too large. Based on the analysis of equivalent plastic strain, simulations were conducted with rolling speeds of 10 m/min and gaps between the upper and lower rolls set at 5 mm, 5.3 mm, and 5.6 mm to determine the force in the X-direction experienced by the rolls. $F_{X}$ is when the positive direction points to the left side of the material’s travel direction; $F_{Y}$ the positive direction is vertically upward; $F_{Z}$ the positive direction is in the direction of material travel; $F_{n}$ the resultant force is the combined force in the X-Y-Z axes.

Since the X-direction force for the upper and lower rolls is an axial force, and the forces in the Y and Z directions are bending forces for the roll shaft; this section discusses both $F_{X}$ axial force and $F_{n}$ the combined force in the XYZ directions. 

**Figure 15:**
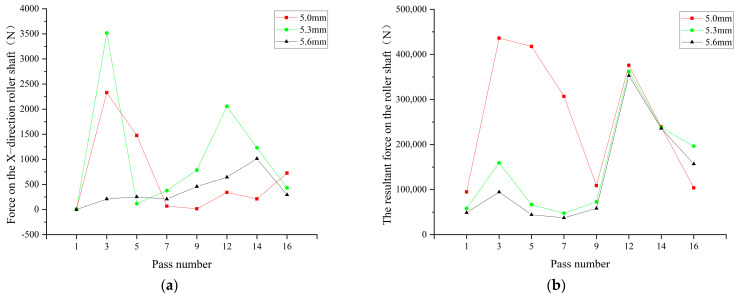
(**a**) Impact of different gaps between upper and lower rolls on the force in the X−direction experienced by the rolls; (**b**) impact of different gaps between upper and lower rolls on the combined force experienced by the rolls.

Simulation data, as shown in Figure 15a, reveal that when the gap between the upper and lower rolls is set to 5.3 mm, the force experienced by the rolls in the X-direction is greater than that of the other two gaps, especially in the 3rd and 12th passes where the force experienced by the rolls increases to 3517.5 N and 2056 N, reaching two peak values. This study found that when the gap between the upper and lower rolls is set to 5.6 mm, the average force in the X-direction is 59.6% and 36.2% of that at gaps of 5.0 mm and 5.3 mm, respectively. This indicates that the axial load on the rolls is minimal at this gap, making it most suitable for processing.

As shown in Figure 15b, when the gap between the upper and lower rolls is changed to 5.3 mm and 5.6 mm, the force experienced by the rolls during the pre-forming stage is significantly less than when the gap is 5.0 mm, while it has a minimal impact on the precision-forming stage. When the gap between the upper and lower rolls is increased from 5 mm to 5.3 mm and 5.6 mm, the combined force experienced by the roll shaft decreases by 42.4% and 50.6%, respectively. This suggests that increasing the gap between the upper and lower rolls can, to some extent, reduce the work hardening during material forming, significantly lowering the combined force or torque experienced by the rolls. This finding provides an important reference for optimizing the material forming process. By adjusting the gap between the upper and lower rolls, it is possible to regulate the torque distribution during the processing, thereby reducing the wear and tear on the equipment, extending its service life, and enhancing the production efficiency of material forming. This can also lower maintenance costs, offering a more reliable guarantee for production. Therefore, appropriately adjusting the gap between the upper and lower rolls can optimize the processing procedure, thereby yielding greater economic benefits. Experiments also show that the combined forces experienced by the rolls during the precision-forming stage are generally higher than those during the pre-forming stage. For example, when the gap between the upper and lower rolls is 5.6 mm, the combined force experienced during the pre-forming stage is only 22.8% of that during the precision-forming stage. This indicates that enterprises should focus on checking and maintaining the roll units during the precision-forming stage in production, repairing and replacing hazardous parts in a timely manner to prevent accidents. 

### 3.4. Analysis of Material Properties of High-Strength Steel 

Based on the experimental conclusions mentioned above, the best forming quality is achieved when the distance between the upper and lower rollers is 5.6 mm and the feed speed is 10 m/min, resulting in a smaller equivalent plastic strain and reduced force on the rollers Therefore, we will analyze the performance characteristics of high-strength steel materials from the aspects of the thickness strain and cross-sectional forming of the plate, in order to verify the advantages of forming high-strength steel plates.

**Figure 16:**
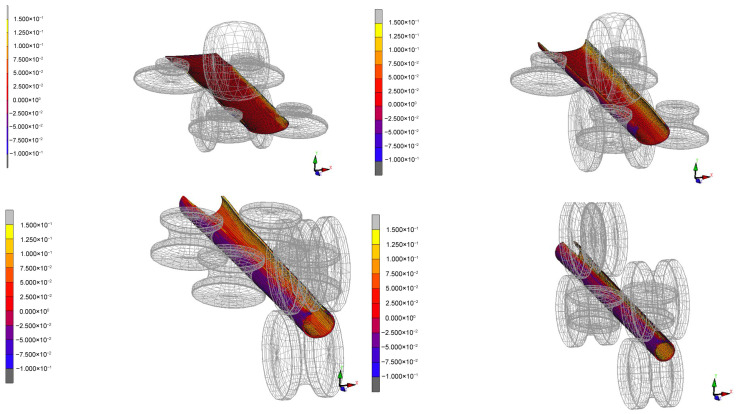
The distribution diagrams of thickness strain for the 4th, 8th, 12th, and 16th forming passes.

As shown in Figure 16, by observing the typical distribution diagram of thickness strain for each forming pass, it is found that for the plate with a lower roller pressure thickness of 5 mm and a distance between the upper and lower rollers of 5.6 mm, due to the high compressive strength of high-strength steel plates, the range of thickness strain is within ±5%. The thickness of the plate is controlled between 4.75 mm and 5.25 mm. The overall deformation of the plate is relatively small, and the strain distribution is uniform. This ensures uniform thickness variation in the plate during the forming process, reduces subsequent stress concentration phenomena, and facilitates subsequent high-pressure internal processing.

**Figure 17:**
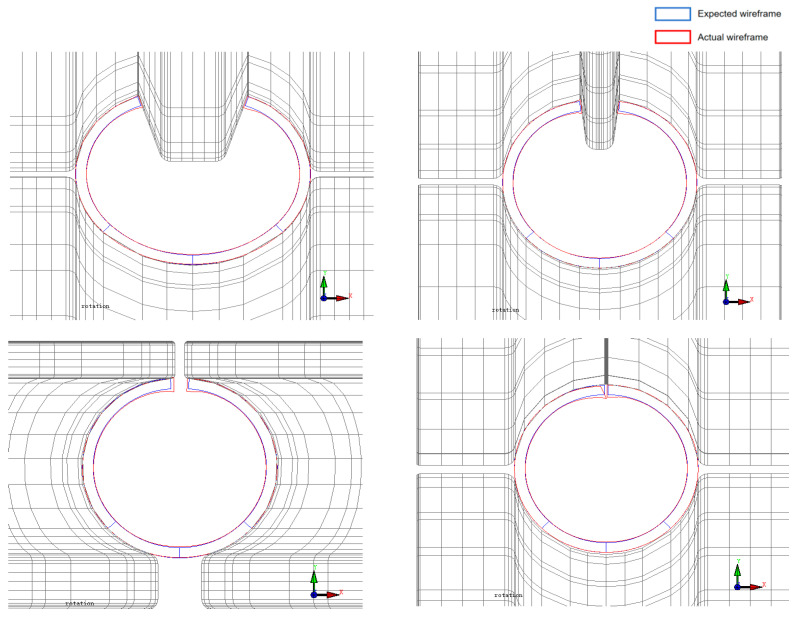
Comparison of cross-sections of precision-forming passes.

As shown in Figure 17, the blue wireframe represents the desired design for sheet metal forming, while the red wireframe represents the actual forming contour. By comparing the cross-sectional forming contours for each forming pass, it is observed that under the influence of high-strength steel materials, the overall deformation of the sheet metal is minimal and closely matches the expected design. Particularly in the precision-forming sections, due to the high tensile strength of high-strength steel, the forming shape of the sheet metal is closer to the desired tubular shape. This reduces the occurrence of defects such as wrinkling and fracturing of the sheet metal while also lowering the difficulty of subsequent welding and improving the quality of sheet metal forming. 

### 3.5. Comparison of Forming Product Results before and after Roller Optimization

**Figure 18:**
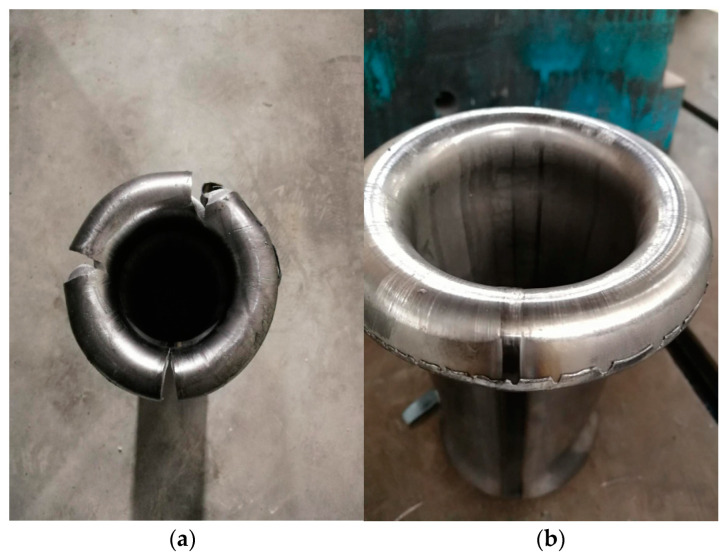
(**a**) Pre-roller optimization arc punching results; (**b**) Post-roller optimization arc punching results.

The results of the tube punching arc are shown in Figure 18, allowing for a clear comparison between the two images. The left image shows a formed tube at a 5.0 mm gap between the upper and lower rolls, where multiple cracks appear as a result of the uneven equivalent plastic strain during the material curling process, leading to varying degrees of work hardening, consistent with the experimental results mentioned above. By adjusting the position of the upper and lower rolls and setting the gap to 5.6 mm in conjunction with the servo feedback for torque and speed matching and fine-tuning the gaps between rolls in other passes based on feedback torque to keep the torque values around 30% for each pass, the results of the adjusted tube punching arc experiment show relatively minor and uniform work hardening. The right image of the punching arc experiment displays excellent punching arc results, qualifying as an acceptable product. The reliability of numerical simulation analysis of roll forming process of high-strength steel is verified.

## 4. Conclusions

This article conducts calculations and comparisons of the lengths of the edge trajectories of sheet metal using four different forming methods: circular bending method, center bending method, edge bending method, and combined bending method. It analyzes the advantages and disadvantages of each forming method and ultimately selects the most suitable forming method for high-strength plates, namely the combined bending method. Based on the comprehensive bending forming method, a forming unit is designed, and numerical simulation analysis of the forming process of high-strength steel longitudinal welded pipes is conducted using finite element analysis software COPRA. The distribution patterns of equivalent plastic strain and work hardening during the forming stages of the sheet metal are obtained, mainly concentrated at the edges and boundary points of the sheet metal. Additionally, it verifies that using high-strength steel for roll forming can reduce the difficulty of subsequent welding, alleviate subsequent stress concentration phenomena, and facilitate subsequent internal high-pressure processing. Addressing forming defects such as excessive equivalent plastic strain, residual stress, and severe work hardening at the edges and boundary points of the high-strength steel longitudinal welded pipes during the forming process, the rollers are optimized. Simulation and analysis are conducted on the effects of different production parameters, such as processing speed during the forming stage and different spacing between upper and lower forming rollers, on the distribution of equivalent plastic strain of the sheet metal and the stress on the rollers. The main conclusions are summarized as follows:

(1) By changing the forming speed of the material in the simulations, the distribution pattern of equivalent plastic strain was analyzed. It was found that processing speed has a minor impact on the distribution of equivalent plastic strain and work hardening in the material, allowing for the processing speed to be reasonably arranged according to production needs.
(2) On the premise of ensuring forming, with the increase in the distance between the upper and lower rollers, the equivalent plastic strain of the sheet metal in the pre-forming stage was reduced by 19.8% at most, while the plastic strain in the fine forming stage was reduced by 6.4%. This shows that adjusting the distance between the upper and lower rollers can improve the plastic strain distribution of the sheet, make the work hardening more uniform, and improve the quality of the formed tube.
(3) Through analyzing the impact of different gaps between the upper and lower rolls on the force experienced by the rolls, it was found that during the material forming process, the forces in the X and Z directions on the rolls are minimal, with the main forces concentrated in the Y direction, i.e., the vertical direction. Additionally, as the gap between the upper and lower rolls increases, the force experienced by the rolls during the pre-forming stage significantly decreases while having a minor impact on the precision-forming stage. By adjusting the gap between the upper and lower rolls, the value of the torque applied to the rollers during plate forming is significantly reduced, which can lessen the wear on the equipment and extend its service life, thereby providing greater economic benefits. Furthermore, the combined force experienced by the rolls during the precision-forming stage is significantly greater than during the pre-forming stage. Therefore, enterprises should perform timely maintenance on the roll units during the precision-forming stage to ensure safe production.

## Figures and Tables

**Table 1:** Material property parameters.

Parameters	Values	Units
Poisson’s ratio	0.3	-
Yield strength	750	MPa
Tensile strength	820	MPa
Rate of elongation	18	%
Young’s modulus	750	MPa
Hardening coefficient	0.1	-

**Table 2:** Comparison of the different forming methods.

	Path Length	Rising Height	Bend Times	Forming Stability
Circular bending method	4.44R	2.28R	less	instability
Center bending method	4.49R	2.57R	more	stabilize
Edge bending method	4.0R	2R	more	stabilize
Combined bending method	4.0R~4.44R	2.0R~2.57R	less	stabilize

**Table 3:** Bending angle and radius of the round tube for each pass corresponding to the roll flower pattern diagram.

Pass	$R_{Y(i)}/mm$	$\theta_{ci}/rad$	$\rho_{ci}/mm$	$\theta_{ei}/rad$
1	134.42	0.11	1169.86	0.26
2	132.28	0.16	693.59	0.52
3	128.39	0.18	567.89	0.79
4	122.75	0.17	543.4	1.05
5	115.49	0.13	598.11	1.31
6	106.77	0.08	831.78	1.57
7	96.82	0.19	330.54	1.65
8	85.91	0.30	204.59	1.73
9	74.31	0.39	146.5	1.81
10	62.35	0.48	112.89	1.88
11	50.38	0.56	90.98	1.96
12	38.77	0.62	75.66	2.04
13	27.88	0.68	64.48	2.12
14	18.14	0.72	56.16	2.2
15	9.95	0.74	49.99	2.28
16	3.76	0.74	45.63	2.36
17	0	0.71	42.97	2.43

## Data Availability

Data are contained within the article.
